# A large-scale analysis of refractive errors in students attending public primary schools in Mexico

**DOI:** 10.1038/s41598-023-40810-5

**Published:** 2023-08-19

**Authors:** Marco Antonio Ramírez-Ortiz, Mónica Amato-Almanza, Iván Romero-Bautista, Miguel Klunder-Klunder, Oswaldo Aguirre-Luna, Iryna Kuzhda, Serge Resnikoff, Kristen Allison Eckert, Van Charles Lansingh

**Affiliations:** 1 Ophthalmology Department, Hospital Infantil de México Federico Gómez, Mexico City, Mexico; 2 School of Optometry, UNAM-FES Iztacala, Tlalnepantla, State of Mexico Mexico; 3 Research Division, Hospital Infantil de México Federico Gómez, Mexico City, Mexico; 4 Ophthalmology Department, Ivano-Frankivsk National Medical University, Ivano-Frankivsk, Ukraine; 5 Brien Holden Vision Institute, Sydney, NSW Australia; 6 School of Optometry and Vision Science, University of New South Wales, Sydney, NSW Australia; 7 Independent Consultant, San Antonio Tlayacapan, Jalisco, Mexico; 8 Department of Public Health Sciences, University of Miami Miller School of Medicine, Miami, FL USA; 9 HelpMeSee, New York, NY USA; 10 See Better to Learn Better Foundation, Mexico City, Mexico; 11 Instituto Mexicano de Oftalmología, Av. Armando Birlain Shaffler S/N, Centro Sur, 76090 Santiago de Querétaro, QRO Mexico

**Keywords:** Eye diseases, Refractive errors

## Abstract

A cross-sectional, retrospective study was conducted from September 2013 through July 2014 to determine the prevalence of refractive errors among students attending public primary schools in Mexico. Among 3,861,156 students at 14,566 public primary schools in all 32 states of Mexico, teachers identified reduced visual acuity in 1,253,589 (32.5%) using visual acuity measurement. Optometrists confirmed 391,498 [31.2%, mean (SD) age: 8.8 (1.9) years; 204,110 girls (52.9%)] had refractive errors using visual acuity measurement and noncycloplegic static retinoscopy. Among 288,537 (72.4%) of children with previous eyeglasses usage data reported, 241,505 (83.7%) had uncorrected refractive errors. Before prescription eyeglasses were provided, 281,891 students (72%) had logMAR visual acuity ≤ 0.2; eyeglasses corrected vision loss in 85.6% (n = 241,352) of them. Simple myopic astigmatism was the most frequent refractive error (25.7%, n = 100,545). Astigmatism > − 1.00 diopters was present in 54.6% of all students with ametropia. The anisometropia rate based on spherical equivalent difference between right and left eye ≥ 1.50 diopters was 3.9% (n = 15,402). Uncorrected refractive errors are an important issue in primary school students in Mexico. An updated study is needed to analyze the evolving trends over the past decade.

## Introduction

Refractive errors, including myopia, hyperopia, astigmatism, and presbyopia, are very common eye disorders that occur when the eye's optical system cannot sharply focus images, causing blurred vision^1^. Uncorrected refractive errors (URE) are the leading cause of moderate and severe vision impairment and the third leading cause of blindness in adults aged 50 years and older^2^. Data are limited for the pediatric population. A 2017 systematic review and meta-analysis estimated that among the global population younger than 20 years, 11.7% [95% Confidence Interval (CI): 10.5–13.0) had myopia, 4.6% (95% CI 3.9–5.2) had hyperopia, and 14.9% (95% CI 12.7–17.1) had astigmatism^3^. A more recent pooled analysis published in 2022 estimated that the prevalence of URE among the population younger than 20 years was 5.85 per 1,000 (95% Confidence Interval: 3.75–9.13) in the Americas (including the United States)^4^. By 2050, up to half the world population could have myopia^5^. Refractive errors can be easily diagnosed by ocular examination and should be treated with eyeglasses or other refractive corrections^1^. Their diagnosis and treatment are among the easiest and more cost-effective ways to reduce vision impairment and even blindness^1,6,7^.

In children, URE are known to affect school performance and can result in early school leaving, generating loss of individual, family, and social opportunities and reducing productivity^7–14^. School screening programs can identify, refer, and facilitate treatment of children with URE. Studies have shown that the provision of free school-based vision screening with free eyeglasses to appropriate students improves academic performance^11,15,16^.

In Mexico, there have been no large-scale, countrywide studies on the impact of refractive errors in children. In 2010, the National Institute of Statistics and Geography (*Instituto Nacional de Estadística, Geografía e Informática* hereafter referred to by its Spanish acronym, INEGI) reported that approximately 800,000 out of 67 million primary school-aged students in the country (1.2%) had some degree of vision impairment^17^. The See Better to Learn Better Vision Program (*Ver Bien Para Aprender Mejor;* hereafter referred to by its Spanish acronym, VBAM) is a public–private educational organization in Mexico that has collaborated with the Ministry of Education of Mexico since 1998 to provide free eyeglasses and comprehensive vision care to school children. Every year, program optometrists examine students in public schools throughout the country. The program also trains teachers to detect possible vision problems through a gross detection visual acuity eye exam. The aim of this study was to determine the prevalence of refractive errors among students attending public primary schools in Mexico.

## Methods

According to INEGI, in 2010, there were 79,480 public primary schools in Mexico^18^. This retrospective study analyzed student data collected from a cross-sectional, random sample of 14,566 (18.3%) public primary schools in 136 municipalities in all 32 states of Mexico from September 2013 through July 2014; these dates correspond to the start and end of the Mexican academic school year. There was no predetermined sample size calculated. The study sample was determined in each Mexican State by the Ministry of Education local Office of Planning and Coordination, which provided VBAM with a list of public primary schools. Trained schoolteachers provided free visual acuity eye examinations and distributed free eyeglasses prescribed by optometrists, following the provision of consent from school authorities. Schoolteachers obtained written informed consent from parents and legal guardians of all students who received eye exams, in accordance with the Ministry of Education laws. The Ethics and Research Committee of See Better to Learn Better approved the study protocol, which was conducted in accordance with the ethical principles of the Declaration of Helsinki.

There were 4 phases of this study. During Phase 1, all students aged 6 to 12 years attending grades 1 through 6 in public primary schools, whose parents/guardians provided informed consent, were evaluated.

The following student information was collected at each visit: age, sex, municipality, state of residence, presenting monocular and binocular visual acuity (with limited children wearing refractive correction when examined), refractive error found (sphere, negative cylinder, and axis), and if they wore eyeglasses on the day of the visit. During Phase 1, school authorities scheduled visual acuity examinations to take place in well-illuminated classrooms during school hours, in such a manner as not to disrupt the daily routine^19^. Teachers used HOTV charts (Precision Vision, Woodstock, IL) to measure binocular visual acuity at a distance of 6 m. Following the recommendation of the VISIONS 2020: The Right to Sight Program that binocular assessment should be used in public health screening^20^, VBAM considered binocular assessment more appropriate for school teachers in a gross screening environment, as monocular assessment takes much longer during busy school hours in a distracting environment, and teachers are not skilled enough to properly cover 1 eye without blurring vision. Children with binocular visual acuity of 20/32 who passed the gross screening visual exam conducted by teachers were excluded from the study. Children whose parents failed to sign an informed consent form were also excluded. Those students who failed the 20/32 visual acuity threshold were referred to the certified optometrists for comprehensive eye exams in Phase 2, which consisted in measuring monocular and binocular presenting visual acuities with logMAR standardized eye charts at 6 m and static retinoscopy^21^. Static retinoscopy without cycloplegic agents and cross cylinder subjective tests were applied during refractive examination. The spherical equivalent (SE) of each student was obtained, defined as the sum of the sphere and half of the cylinder (sphere + ½ cylinder). Spherical refraction was recorded for both eyes. Emmetropia was defined as a SE of ≥ − 0.50 to ≤  + 0.50 diopter (D) sphere in both eyes. Astigmatism was defined as a cylindrical error of ≤ − 0.50 D cylinder at any axis^22^. Five different types of astigmatism were captured: (1) compound hyperopic astigmatism, defined as when both main meridians of the eye have hyperopic refractions of different degrees; (2) simple hyperopic astigmatism, when one meridian of the eye has emmetropia and the other has hyperopic refraction; (3) mixed astigmatism, when 1 meridian is myopic and the other is hyperopic; (4) compound myopic astigmatism, when both meridians have myopic refraction of different degrees; and (5) simple myopic astigmatism, when 1 meridian is emmetropic and the other is myopic^23^. Anisometropia was defined as a difference in SE of ≥ 1.00 D^24^.

Definitions of vision impairment and blindness were based on distance presenting visual acuity (mild vision impairment: < 6/12 to 6/18), moderate: < 6/18 to 6/60, severe: < 6/60 to 3/60, and blindness < 3/60)^25^. During Phase 3, eyeglass prescriptions were provided at clinician discretion. Eyes with hyperopic SE ≥  + 2.00 D, myopic SE ≤ − 0.50, or astigmatism > − 0.50 D were recommended for eyeglass prescription^17,26^. Eyeglasses were prescribed based on subjective refractions, providing full correction of astigmatism and either full correction or symmetrical under correction of hyperopia by no more than + 1.50 D. During Phase 4, fully customized eyeglasses were manufactured and freely provided to students identified in Phase 3.

All study data were collected in Excel spreadsheets. Statistical analyses were done using Intercooled Stata 16 SE (StataCorp, College Station, TX). The population was divided into 3 age groups for analysis: 6 to 8 years old, 9 to 10 years old, and 11 to 12 years old. The country was divided into 8 regions (Northeast, Northwest, West, East, North-Central, South-Central, Southeast, and Southwest), according to the National Health Survey of 2012^27^. Analyses were done on the right eye, per-person basis (except when indicated), because refractive errors were symmetrically distributed both in right eye and left eye in our study population (Pearson correlation test r = 0.895). Descriptive statistics were performed using mean and standard deviation (SD) for continuous measures. Categorical variables are expressed in frequency count and percentage. Comparisons of proportions were made with the *X*^2^ test and comparisons of means with Student’s t test. The frequency of vision impairment caused by refractive errors was based on the prevalence of current visual acuity < 6/18 (20/60) in the better eye, according to WHO guidelines^28^. The distribution of right eye noncycloplegic SE (D) for students who failed school vision screening was tested for normal distribution using skewness and kurtosis, by age group: 6–8 years, 9–10 years, 11–12 years old, and in all ages. All statistical tests were two-sided, and *p* < 0.01 was considered statistically significant.

## Results

**Figure 1:**
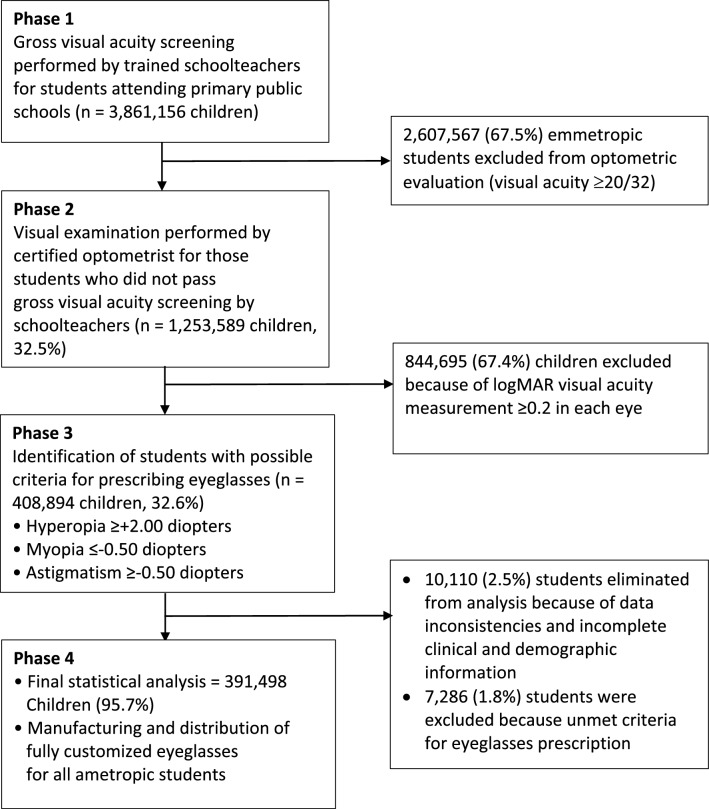
Participant flow diagram.

During the 2013–2014 school year and Phase 1 of the study, school screenings were conducted on 3,861,156 students in public primary schools of Mexico; 2,607,567 (67.5%) were excluded from the study due to teachers reporting that they had a visual acuity ≥ 20/32. Figure 1 summarizes the participant flow. During Phase 2, certified optometrists performed comprehensive eye exams on 1,253,589 (32.5%) students with possible visual acuity problems and identified 408,894 students with possible refractive errors in Phase 3. Due to incomplete or inconsistent data reported, 10,110 students from the state of Chihuahua were excluded from analysis. Another 7,286 students were withdrawn, because the optometrists did not follow the prescription criteria and recommended students who should have been excluded in Phase 2, because they were emmetropic with very mild refractive error that did not require correction.. During Phase 4, customized eyeglasses were manufactured and distributed to all students with significant refractive error. Final statistical analysis was performed on 391,498 children (Fig. 1). Among the study population analyzed, their mean age was 8.8 years (range: 6–12 years, SD: 1.9), and the majority (52.9%, n = 204,110) were girls.

Table 1 summarizes the prevalence of refractive errors in the student population. In relation to SEs, there were no differences between the right eye and left eye. More than 70% of SE refractions were located between + 1.50 and − 1.50 D, with non-normal distribution with a slight amount of kurtosis (14.8 for all ages) and skewness (− 1.61 for all ages) towards negative SE. This distribution trend was similarly observed in the 6–8 year age group (skewness: − 1.43; kurtosis: 15.4), the 9–10 year age group (− 1.64 14.8), and the 11–12 year age group (− 1.78; 14.7), with all groups demonstrating leptokurtic distribution (*p* < 0.001). The most common refractive error was simple myopic astigmatism, observed in 25.7% of all students (n = 100,545), followed by mixed astigmatism found in 21.8% of students (n = 85,330). As for cylindrical errors, moderate astigmatism (− 1.00 to − 2.75 D) was the most frequently found in 37.5% of students (n = 146,664), and 17.1% of the cylinders (n = 67,071) were greater than − 3.00 D (Table 1).

**Table 1:** Demographic and clinical characteristics of public primary school students with significant refractive errors disaggregated by sex (n = 391,498).

	Female (n = 204,110)		Male (n = 187,388)		Total (n = 391,498)	
	n	%	n	%	n	%
Ages, years						
6–8	84,781	41.5	80,189	42.8	164,970	42.1
9–10	68,476	33.5	61,305	32.7	129,781	33.1
11–12	50,853	24.9	45,894	24.5	96,747	24.7
Region						
Northeast	35,667	17.5	30,156	16.1	65,823	16.8
Northwest	35,597	17.4	31,593	16.9	67,190	17.2
West	30,634	15	28,176	15	58,810	15
East	20,347	10	19,197	10.2	39,544	10.1
North Central	32,297	15.8	29,829	15.9	62,126	15.9
South Central	29,033	14.2	28,230	15.1	57,263	14.6
Southwest	11,985	5.9	11,329	6.1	23,314	6
Southeast	8,550	4.2	8,878	4.7	17,428	4.4
Right eye refractive error						
Compound hyperopic astigmatism	27,959	13.7	23,648	12.6	51,607	13.2
Simple hyperopic astigmatism	7,978	3.9	7,227	3.9	15,205	3.9
Mixed astigmatism	42,358	20.8	42,972	22.9	85,330	21.8
Compound myopic astigmatism	27,527	13.5	23,794	12.7	51,321	13.1
Simple myopic astigmatism	49,303	24.2	51,242	27.4	100,545	25.7
Hyperopia	23,381	11.5	17,402	9.3	40,783	10.4
Myopia	25,604	12.5	21,103	11.3	46,707	11.9
Cylinder right eye						
0 to −0.25	58,063	28.4	45,051	24	103,114	26.3
−0.50 to −0.75	40,928	20	33,721	18	74,649	19.1
−1.00 to −2.75	72,344	35.4	74,320	39.7	146,664	37.5
> − 3.00	32,775	16.1	34,296	18.3	67,071	17.1

**Figure 2:**
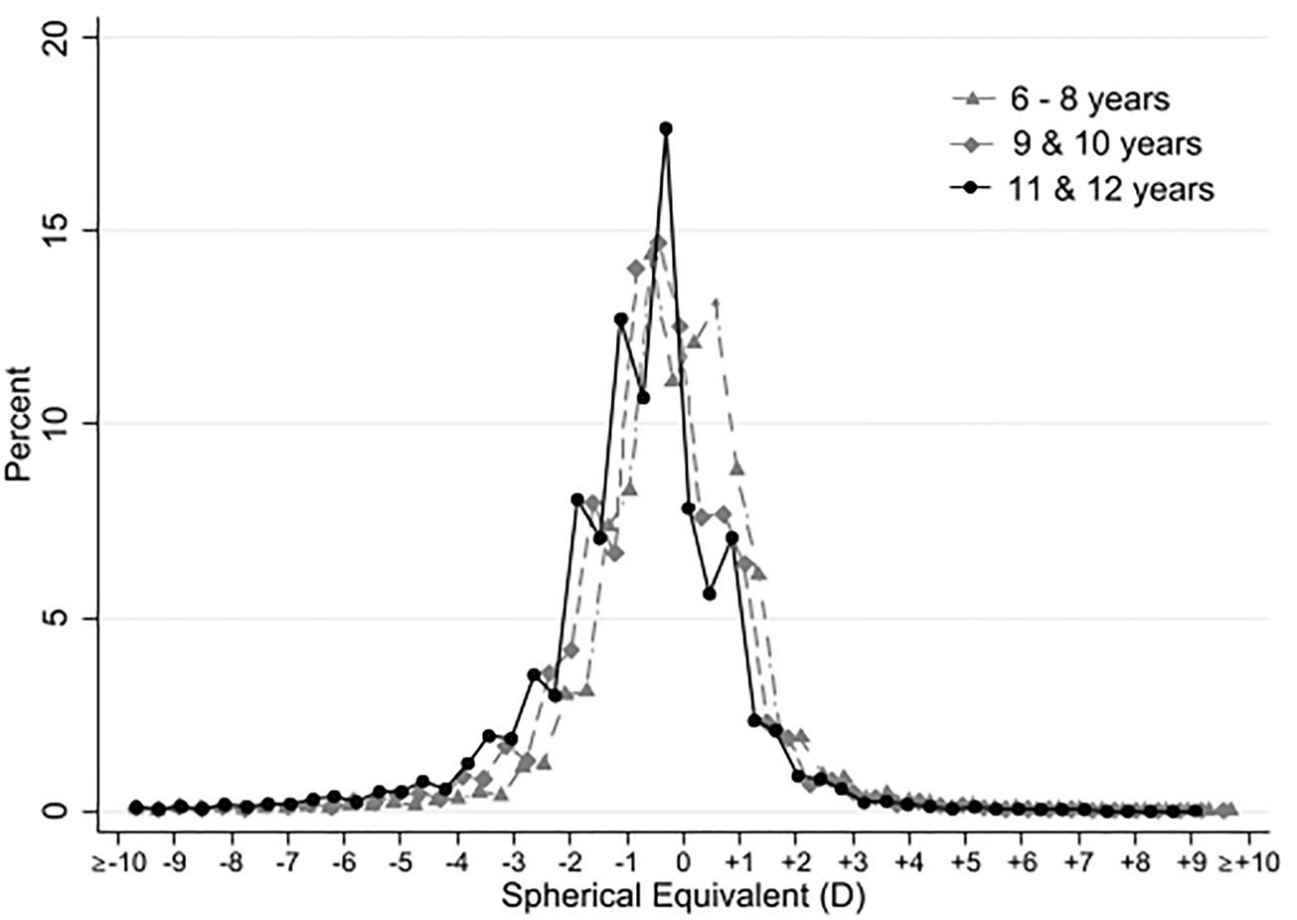
Distribution of right eye noncycloplegic spherical equivalent in diopters (D) for students who failed school vision screening, by age group: 6–8 years, 9–10 years, and 11–12 years old.

Table 2 summarizes the distribution of significant refractive errors across the 8 regions of Mexico. The differences in all values across the regions were statistically significant (*p* < 0.001). Mixed astigmatism was more frequent in the southern regions, whereas hyperopic astigmatism was more frequent in the Northeast (Table 2 and Fig. 2).

**Table 2:** Distribution of significant refractive errors among public primary school students by region in Mexico (n = 391,498). T test of differences for all values were statistically significant (p < 0.001).

Region	Compound Hyperopic Astigmatism		Simple Hyperopic Astigmatism		Mixed Astigmatism		Compound Myopic Astigmatism		Simple Myopic Astigmatism		Hyperopia		Myopia		Total	
	n	%	n	%	n	%	n	%	n	%	n	%	n	%	n	%
Northeast	12,022	23.3	3,044	20	12,632	14.8	6,829	13.3	14,058	14	10,400	25.5	6,838	14.6	65,823	16.8
Northwest	9,543	18.5	2,562	16.8	13,480	15.8	8,969	17.5	14,649	14.6	7,952	19.5	10,035	21.5	67,190	17.2
West	8,754	17	2,235	14.7	13,530	15.9	8,100	15.8	13,158	13.1	6,002	14.7	7,031	15	58,810	15
East	4,257	8.2	1,503	9.9	10,095	11.8	4,967	9.7	12,014	11.9	2,337	5.7	4,371	9.4	39,544	10.1
North Central	7,587	14.7	2,541	16.7	15,016	17.6	8,428	16.4	16,325	16.2	5,407	13.2	6,822	14.6	62,126	15.9
South Central	4,644	9	1,771	11.6	12,492	14.6	9,342	18.2	19,114	19	3,689	9	6,211	13.3	57,263	14.6
Southwest	2,921	5.7	898	5.9	4,878	5.7	2,503	4.9	6,382	6.4	3,295	8.1	2,437	5.2	23,314	6
Southeast	1,879	3.6	651	4.3	3,207	3.8	2,183	4.2	4,845	4.8	1,701	4.2	2,962	6.3	17,428	4.4
Total	51,607	100	15,205	100	85,330	100	51,321	100	100,545	100	40,783	100	46,707	100	391,498	100

Table 3 summarizes the available visual acuity of the worst eye before and after prescription eyeglasses were provided to students with refractive errors. Presenting visual acuity in the worst eye lower than 0.2 logMAR (Snellen < 20/30) was present in 72% (n = 281,891) of all children evaluated by optometrists; after best correction with eyeglasses, only 40,919 students (10.4%) remained in this category, and only 7,628 (1.9%) still had visual acuities worse than 0.5 logMAR (≤ 20/80).

**Table 3:** Visual acuity distribution (logMAR) in the worst eye before and after prescription eyeglasses were provided to 391,498 public primary school students with refractive errors.

Vision Impairment Category	Presenting Binocular Distance Visual Acuity	No. of Students before Eyeglass Prescription (%)	No. of Students after Eyeglass Prescription (%)	Change in No. of Students (%)
None	0.2 or better	102,320 (26.1)	343,672 (87.8)	+ 241,352 (+ 235.9)
Mild	0.3 to 0.4	133,766 (34.2)	33,291 (8.5)	− 100,457 (− 75.1)
Moderate	0.5 to 1.0	138,764 (35.4)	6,492 (1.7)	− 132,272 (− 95.3)
Severe	1.1 to 1.3	770 (0.2)	7 (0)	− 763 (− 99.1)
Blindness	1.4 or worse	8,591 (2.2)	1,129 (0.3)	− 7,462 (− 86.9)
Missing data	N/A	7,287 (1.9)	6,907 (1.8)	− 380 (− 5.2)

N/A = Not applicable.

Table 4 summarizes the prevalence of anisometropic refractions. The overall frequency of anisometropia ≥ 1.00 D was found in 30,845 students (7.9%) and anisometropias > 1.50 D were found in 15,402 (3.9%).

**Table 4:** Summary of prevalence of differences of anisometropia based on spherical equivalent (SE) in public primary school students, by age group (n = 391,498).

SE Difference	6–7 years, n (%)	8–10 years, n (%)	11–12 years, n (%)	All Ages, n (%)
≥ 1.00	11,138 (6.8)	10,750 (8.3)	8,957 (9.2)	30,845 (7.9)
≥ 1.50	5,319 (3.2)	5,359 (4.1)	4,724 (4.9)	15,402 (3.9)

SE = Spherical equivalent.

Hyperopic SE ≥ 2.00 D was more frequent in the 6–8 years age group, whereas myopic SE ≤ − 0.50 D was more frequent in the 11–2 years age group (Table 5). We found no significant SE differences between sexes in any age groups (Table 5; Fig. 2; Supplementary Figs. S1A and S1B).

**Table 5:** Distribution of right eye noncycloplegic spherical equivalents in diopters (D) for students who failed school vision screening, disaggregated by age groups and by sex, n (%).

D	Ages 6 – 8 years			Ages 9 – 10 years			Ages 11–12 years			Ages 6 – 12 years		
	F	M	Both	F	M	Both	F	M	Both	F	M	Both
≥ − 10	228 (0.3)	201 (0.2)	429 (0.3)	272 (0.4)	223 (0.4)	495 (0.4)	320 (0.6)	227 (0.5)	547 (0.6)	820 (0.4)	651 (0.4)	1,471 (0.4)
≥ − 9	109 (0.1)	69 (0.1)	178 (0.1)	124 (0.2)	98 (0.2)	222 (0.2)	100 (0.2)	90 (0.2)	190 (0.2)	333 (0.2)	257 (0.1)	590 (0.2)
≥ − 8	134 (0.2)	141 (0.2)	275 (0.2)	158 (0.2)	127 (0.2)	285 (0.2)	147 (0.3)	112 (0.2)	259 (0.3)	439 (0.2)	380 (0.2)	819 (0.2)
≥ − 7	155 (0.2)	159 (0.2)	314 (0.2)	213 (0.3)	172 (0.3)	385 (0.3)	243 (0.5)	165 (0.4)	408 (0.4)	611 (0.3)	496 (0.3)	1,107 (0.3)
≥ − 6	261 (0.3)	202 (0.2)	463 (0.3)	332 (0.5)	249 (0.4)	581 (0.4)	395 (0.8)	268 (0.6)	663 (0.7)	988 (0.5)	719 (0.4)	1,707 (0.4)
≥ − 5	397 (0.5)	304 (0.4)	701 (0.4)	531 (0.8)	367 (0.6)	898 (0.7)	650 (1.3)	426 (0.9)	1,076 (1.1)	1,578 (0.77)	1,097 (0.6)	2,675 (0.7)
≥ − 4	629 (0.7)	519 (0.7)	1,148 (0.7)	950 (1.3)	728 (1.2)	1,678 (1.3)	1,089 (2.1)	858 (1.9)	1,947 (2)	2,668 (1.3)	2,105 (1.1)	4,773 (1.2)
≥ − 3	1,207 (1.5)	1,156 (1.4)	2,426 (1.5)	2,186 (3.2)	1,650 (2.7)	3,836 (3)	2,230 (4.4)	1,811 (4)	4,401 (4.2)	5,686 (2.8)	4,617 (2.5)	10,303 (2.6)
≥ − 2	4,100 (4.8)	3,921 (4.9)	8,021 (4.9)	5,716 (8.4)	5,246 (8.6)	10,962 (8.4)	5,575 (11)	4,979 (10.8)	10,554 (10.9)	15,391 (7.5)	14,146 (7.6)	29,537 (7.5)
≥ − 1	13,903 (16.4)	14,287 (17.8)	28,190 (17.1)	14,418 (21.1)	13,981 (22.8)	28,399 (21.9)	11,852 (23.3)	11,561 (25.2)	23,413 (24.2)	40,173 (19.7)	39,829 (21.2)	80,002 (20.4)
0	45,567 (53.8)	43,152 (53.8)	88,719 (53.8)	34,018 (49.7)	29,835 (48.7)	63,853 (49.2)	22,764 (44.8)	20,441 (44.5)	43,205 (44.7)	102,349 (50.1)	93,428 (49.9)	195,777 (50)
≥ 1	13,673 (16.1)	11,330 (14.1)	25,003 (15.2)	7,065 (10.3)	5,769 (9.4)	12,834 (9.9)	4,086 (8)	3,195 (7)	7,281 (7.5)	24,824 (12.2)	20,294 (10.8)	45,118 (11.5)
≥ 2	2,695 (3.2)	2,659 (3.3)	5,354 (3.2)	1,537 (2.2)	1,585 (2.6)	3,122 (2.4)	839 (1.7)	1,005 (2.2)	1,844 (1.9)	5,071 (2.5)	5,249 (2.8)	10,320 (2.6)
≥ 3	919 (1.1)	1,114 (1.4)	2,033 (1.2)	539 (0.8)	642 (1)	1,181 (0.9)	339 (0.7)	390 (0.9)	729 (0.8)	1,797 (0.9)	2,146 (1.2)	3,943 (1)
≥ 4	386 (0.5)	473 (0.6)	859 (0.5)	240 (0.4)	325 (0.5)	565 (0.4)	113 (0.2)	187 (0.4)	300 (0.3)	739 (0.4)	985 (0.5)	1,724 (0.4)
≥ 5	183 (0.2)	237 (0.3)	420 (0.2)	100 (0.2)	150 (0.2)	250 (0.2)	53 (0.1)	93 (0.2)	146 (0.2)	336 (0.2)	480 (0.3)	816 (0.2)
≥ 6	95 (0.1)	151 (0.2)	246 (0.2)	38 (0.1)	86 (0.1)	124 (0.1)	30 (0.1)	45 (0.1)	75 (0.1)	163 (0.1)	282 (0.2)	445 (0.1)
≥ 7	42 (0)	66 (0.1)	108 (0.1)	20 (0)	39 (0.1)	59 (0)	7 (0)	17 (0)	24 (0)	69 (0)	122 (0.1)	191 (0)
≥ 8	14 (0)	25 (0)	39 (0)	8 (0)	9 (0)	17 (0)	5 (0)	8 (0)	13 (0)	27 (0)	42 (0)	69 (0)
≥ 9	4 (0)	11 (0)	15 (0)	6 (0)	10 (0)	16 (0)	4 (0)	4 (0)	8 (0)	14 (0)	25 (0)	39 (0)
≥ 10	17 (58.6)	12 (41.4)	29 (100)	5 (0)	14 (0)	19 (0)	12 (0)	12 (0)	24 (0)	34 (0)	38 (0)	72 (0)

F = Female, M = Male.

Eyeglass usage data was collected from 288,537 (73.7%) children. On the day of examination, 47,023 students (16.3%) were already wearing eyeglasses to correct their refractive errors. Thus, the overall prevalence of URE among the study population was suggested to be 83.7%. All students with refractive error were provided with free eyeglasses in this study, regardless if they were wearing eyeglasses on the day of examination.

## Discussion

In this large, countrywide study of refractive errors in primary school students, 31.2% of students who had vision loss identified from school vision screenings received a confirmatory diagnosis of refractive error (Fig. 1), and prescription eyeglasses corrected vision loss in 85.6% of the affected population (Table 3). The prevalence of severe vision impairment and blindness among the student population before ametropic correction with eyeglasses was 2.4%, which was double the pediatric vision loss estimate of the INEGI in 2010^15^. After eyeglass prescription, only 0.3% had severe vision impairment and blindness (Table 3). Only 16.3% of students with refractive errors were already wearing eyeglasses at school at the time of this study, indicating that URE is a major issue in Mexican and schools, with a troubling gap in eyeglass usage among children, and suggesting that many public primary school students may benefit from vision screening and eyeglass donation.

More than half of the refractive errors in our study were caused by astigmatism (Table 1). Simple myopic astigmatism was the most frequent refractive error found in 25.7% of our study population and was more common in southern Mexico (Tables 1 and 2; Fig. 2). Myopia was only found in 11.9% of the study population, which was nearly equivalent to the 11.7% rate reported in the 2017 global systematic review and meta-analysis^3^, but a much lower rate than those reported in other Mexican studies. A 2005 study of 1,136 students aged 6 to 15 years from the central state of Mexico reported a myopia prevalence rate of 33%^28,29^. In the southern state of Oaxaca in 2006, among 493 students aged 5 to 18 years old, a staggering 74.5% had myopia^19^. Much lower prevalence rates were reported from the most recent Mexican studies. Among 2,647 students aged 5 to 14 years in Quintana Roo in 2014, only 4.6 had myopia^30^. Among 722 pediatric patients of the public health system of Aguascalientes in 2018, 7.0% had myopia^31^. The great differences in prevalence rates of myopia could have resulted from different definitions of myopia used in the Mexican studies^32^. Multiple studies have demonstrated that a slight change in the threshold definition by 0.25 D can significantly affect the prevalence rates^32–35^. Furthermore, unlike in the other smaller Mexican studies, in our study, vision screenings were held at nearly 20% of all public primary schools in Mexico, which were attended by 90% of primary school-aged children during the 2013–2014 academic year^36^. Thus, our findings were more generalizable and highly representative of the target population of Mexico.

The overall anisometropia ≥ 1.00 D rate was 7.9% in our study; 3.9% of students had > 1.50 D (Table 4). Recent studies reported similar prevalence rates using the 1.50 D threshold. In Portugal, the prevalence rate was 6.1% among 749 students aged 3 to 16 years during the 2018–2019 academic year^37^. In France, the prevalence rate was 5.0% among 48,163 children^38^. Anisometropia at a child's first clinical examination has been associated with a high risk of amblyopia^39^, and so, these combined findings highlight the importance of preschool vision screening programs to identify and treat early cases of amblyopia.

Globally, we should be concerned over the increasing rates of refractive errors among children, largely fueled by the SARS-CoV-2 (COVID-19) pandemic lockdown measures. In 2020, school closures and remote learning affected an estimated 1.37 billion students^40^. Multiple systematic reviews and meta-analyses, albeit of predominantly Asian studies, have reported a rapid increase and/or progression in myopia among school-aged children since the pandemic onset, largely due to the increased use of digital devices during remote learning and decreased outdoor activity^41–44^. Studies have similarly reported a 1.5-fold increase in astigmatism among children following school closures^45,46^. Unlike in our prepandemic study, COVID-19 lockdown studies have reported gender inequities in refractive errors among students. Among 3,850 public school students in Southern India, there was a 3- to 6-fold increase in myopia after the lockdown, which was more predominant in girls than boys, likely due to traditional gender roles resulting in girls being even less likely than boys to spend time outdoors and more likely to be perform household chores and have increased screen time^47^. There are no pre- vs postpandemic refractive error data for students in Mexico, and thus, an immediate follow-up study is warranted to see how remote learning, increased use of digital devices, and decreased outdoor activities have affected the refractive error status of Mexican students.

The major limitation of this study is that the data are from the 2013 and 2014 and therefore may not reflect the current situation of refractive error in primary school students, especially after prolific reports from all over the world cite the increase in refractive error among children following the COVID-19 pandemic. We received delayed authorization to publish the study data, and then, the manuscript was further delayed by the COVID-19 pandemic. Nonetheless, it is important to publish these older data to provide baseline data and a historic understanding of the state of refractive error in children before the pandemic. At the time of writing, VBAM is preparing a follow-up analysis to report the prevalence of refractive error in primary school students in Mexico during the 2023–2024 academic year and analyze the refractive error trends over the past decade.

Another major limitation of this study was that the gold standard cycloplegic refraction was not performed^32^. Thus, the prevalence of high hyperopia (only 4.5% in 17,865 students) may have been underestimated (Table 5), although it was very similar to the global prevalence rate of 4.6% reported in the prepandemic, global systematic review and meta-analysis^3^. Furthermore, the previously mentioned analysis of refractive errors in 48,163 children in France used cycloplegic refraction and reported an even lower prevalence of high hyperopia at 3.6%^38^. Similarly, the prevalence of myopia (although quite low at 11.9%) may have been overestimated in this study due to the use of noncycloplegic refraction (Table 1). Noncycloplegic refraction is known to have no significant effect on identifying astigmatism^45,48,49^.

Other study limitations include the lack of a follow-up period; we do not know if students continued to wear their eyeglasses after the study ended, nor do we know the long-term impact of eyeglass provision on students’ activities of daily living, academic performance, or refractive status. While data on effective refractive error coverage are now being collected and reported for adults aged 50 years and older, with global coverage reported to be 20.5% (95% CI 17.8–24.4) and Latin American coverage reported to be 34.5% (95% CI 29.4–40.0)^50^, an effective coverage indicator is not reported for children^51^. However our finding that only approximately 16.3% of children wore eyeglasses at the time of examination aligns with the 2006 Oaxaca study, which reported 13% of students with refractive errors wearing eyeglasses as the time of examination^19^, as well as a more recent Latin American study from Chile, where 14% (144/1,017) of the students with refractive error in at least 1 eye wore eyeglasses at the time of examination^52^. More research on eyeglass usage and compliance among children is urgently needed from the region. While we used a traditional visual acuity chart in our study, children with hyperopia and astigmatism may have still been able to read 6/6 (20/20) letters on the chart and therefore could have passed the gross screening exam; also, the chart would not have been effective in capturing children with binocular functional anomalies^53^. Furthermore, as the gross screening examination measure binocular visual acuity, some cases of anisometropia may have been missed.

The causes of vision loss that were not corrected with eyeglasses in 10.4% of participants in this study were not identified; thus, we could not confirm or manage (for example) definitive amblyopia diagnoses.

This study provides historic, baseline data confirming that the prevalence of refractive errors and their related vision loss has been high among primary school students in Mexico. The provision of free eyeglasses to affected students improved vision in most children, highlighting the importance of free school vision screenings and eyeglass provision to manage URE. Given the toll of the COVID-19 pandemic, including school closures, remote learning measures, and a general widespread increase in dependence and usage of digital devices among Mexican students, a 10-year follow-up study is urgently needed and in planning stages to assess the evolving trends and current burden of refractive errors among primary school students in Mexico. Future investigations should also analyze eyeglass usage and compliance, as well as changes in academic performance among students with refractive errors in Mexico to understand the long-term benefit of school vision programs.

### Supplementary Information

Supplementary Figures.

## Data Availability

The datasets generated and/or analyzed during this study are available from the corresponding author on reasonable request.
