# Supplementary material for: A large-scale analysis of refractive errors in students attending public primary schools in Mexico
Source: Sci Rep. 2023 Aug 19;13:13509. doi: 10.1038/s41598-023-40810-5 (PMC10439951; doi:10.1038/s41598-023-40810-5)
Supplement: Supplementary file 1 — Supplementary Figures. [file 41598_2023_40810_MOESM1_ESM.pdf]

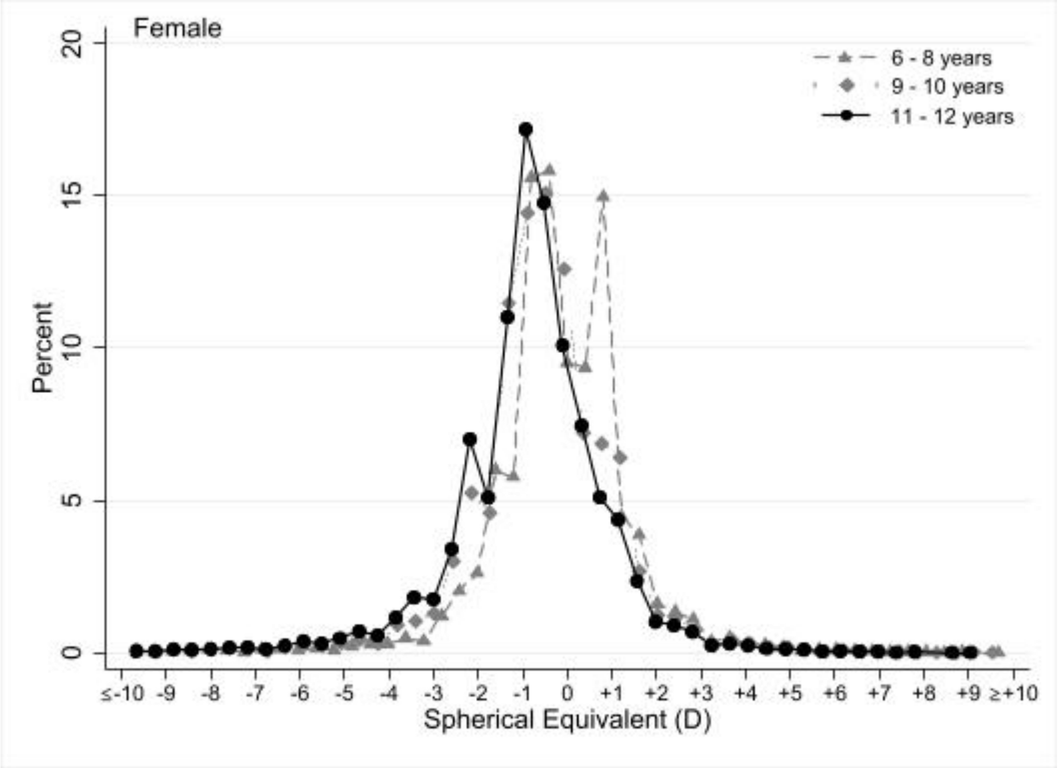

Supplementary Figure 1A. Distribution of right eye noncycloplegic spherical equivalent in diopters (D) for female students who failed school vision screening by age groups: 6–8 years, 9–10 years, and 11–12 years old.

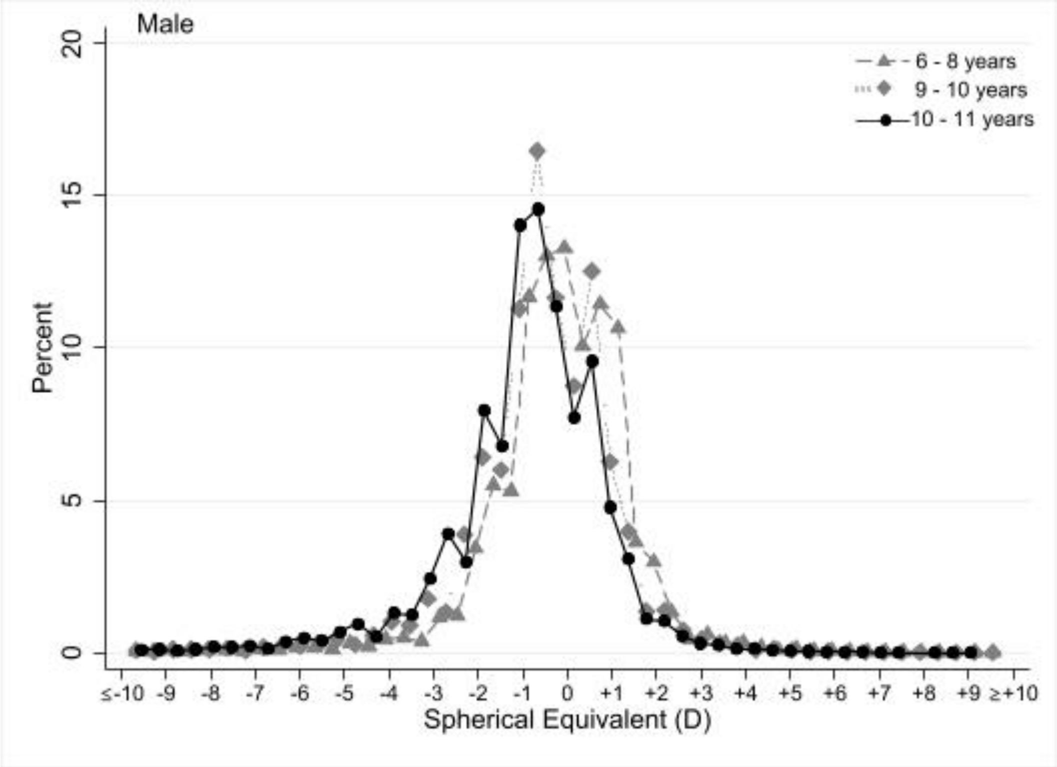

Supplementary Figure 1B. Distribution of right eye noncycloplegic spherical equivalent in diopters (D) for male students who failed school vision screening by age groups: 6–8 years, 9–10 years, and 11–12 years old.
